# Screening immune-related blood biomarkers for DKD-related HCC using machine learning

**DOI:** 10.3389/fimmu.2024.1339373

**Published:** 2024-01-22

**Authors:** Chao Chen, Zhinan Xie, Ying Ni, Yuxi He

**Affiliations:** ^1^ Engineering Research Center of Natural Medicine, Ministry of Education, Advanced Institute of Natural Sciences, Beijing Normal University at Zhuhai, Zhuhai, China; ^2^ Instrumentation and Service Center for Science and Technology, Beijing Normal University at Zhuhai, Zhuhai, China; ^3^ Medical Engineering Department, The Fifth Affiliated Hospital of Sun Yat-sen University, Zhuhai, China; ^4^ Department of Pediatrics, The Second Affiliated Hospital and Yuying Children’s Hospital of Wenzhou Medical University, Wenzhou, China

**Keywords:** DKD-related HCC, immune, machine learning, blood biomarkers, PLVAP

## Abstract

**Background:**

Diabetes mellitus is a significant health problem worldwide, often leading to diabetic kidney disease (DKD), which may also influence the occurrence of hepatocellular carcinoma (HCC). However, the relationship and diagnostic biomarkers between DKD and HCC are unclear.

**Methods:**

Using public database data, we screened DKD secretory RNAs and HCC essential genes by limma and WGCNA. Potential mechanisms, drugs, and biomarkers for DKD-associated HCC were identified using PPI, functional enrichment, cMAP, and machine learning algorithms, and a diagnostic nomogram was constructed. Then, ROC, calibration, and decision curves were used to evaluate the diagnostic performance of the nomograms. In addition, immune cell infiltration in HCC was explored using CIBERSORT. Finally, the detectability of critical genes in blood was verified by qPCR.

**Results:**

104 DEGs associated with HCC using WGCNA were identified. 101 DEGs from DKD were predicated on secreting into the bloodstream with Exorbase datasets. PPI analysis identified three critical modules considered causative genes for DKD-associated HCC, primarily involved in inflammation and immune regulation. Using lasso and RM, four hub genes associated with DKD-associated HCC were identified, and a diagnostic nomogram confirmed by DCA curves was established. The results of immune cell infiltration showed immune dysregulation in HCC, which was associated with the expression of four essential genes. PLVAP was validated by qPCR as a possible blood-based diagnostic marker for DKD-related HCC.

**Conclusion:**

We revealed the inflammatory immune pathways of DKD-related HCC and developed a diagnostic nomogram for HCC based on PLVAP, C7, COL15A1, and MS4A6A. We confirmed with qPCR that PLVAP can be used as a blood marker to assess the risk of HCC in DKD patients.

## 1 Introduction

Diabetes mellitus (DM) has become a significant health problem worldwide, often leading to multiple complications such as diabetic kidney disease (DKD). Multiple factors, such as metabolic disorders due to hyperglycemia and insulin resistance (IR), mitochondrial abnormalities, inflammation, and other factors, play an essential role in the progression of DKD (1). Although DKD is not considered a predominantly “immune-mediated” renal disease, recent experiments in this area suggested that many immune system components are involved in the progression and even initiation of DKD (2). For example, NF-κB is thought to be a major transcription factor in initiating the inflammatory response in DKD, and its inhibition has been shown to ameliorate renal inflammation, oxidative stress, structural damage, and proteinuria (3). Moreover, many other pro-inflammatory signaling pathways are involved in developing DKD, such as inflammatory vesicle activation, mitochondrial DNA (mtDNA) release, and Toll-like receptors (TLRs).

HCC is the fifth most common type of cancer worldwide and the third leading cause of cancer-related deaths worldwide (4). Unlike other malignancies, HCC accounts for approximately 90% of primary liver cancers and primarily develops in the presence of chronic inflammation (5). DKD is a major long-term complication of Type 2 diabetes mellitus (T2DM) associated with chronic inflammation (6). It is unclear whether these systemic chronic inflammations of DKD directly cause HCC. However, Pro-inflammatory cytokines in the pathogenesis of T2DM have been shown to contribute to the development and progression of HCC (7). Moreover, about 50% of T2DM patients develop chronic kidney disease (CKD) (8, 9). Patients with CKD appear to have a higher risk of developing HCC compared to the general population (10). Some studies show that the HCC patient population overlaps highly with the CKD patient population (11). Hence, it is essential to thoroughly examine the communication of these inflammatory factors between DKD and HCC.

Oncogenic signaling receptors associated with the development of HCC appear to be related to DKD-producing molecules. TLR4 and TLR2 play a crucial prognostic role in HCC, associated with HCC occurrence, invasion, and metastasis (12). Uncontrolled TLR-regulated tissue repair responses can drive tumor growth and progression in a positive feedback of uncontrolled tissue injury and repair that triggers a TLR-dependent inflammatory response (13). Moreover, hepatic TLR2 and TLR4 can trigger an inflammatory response by responding to endogenous host molecules known as damage-associated molecular patterns (DAMP), which include oxidized LDL (14), advanced glycosylation end products (AGE) (15), and HMGB1 (16), that are released and elevated during inflammatory conditions like DKD. Therefore, further investigation of the correlation between cytokine-mediated inflammation between DKD and HCC will broaden our understanding of HCC pathogenesis and treatment.

## 2 Materials and methods

### 2.1 Microarray data collecting and parsing

**Figure 1:**
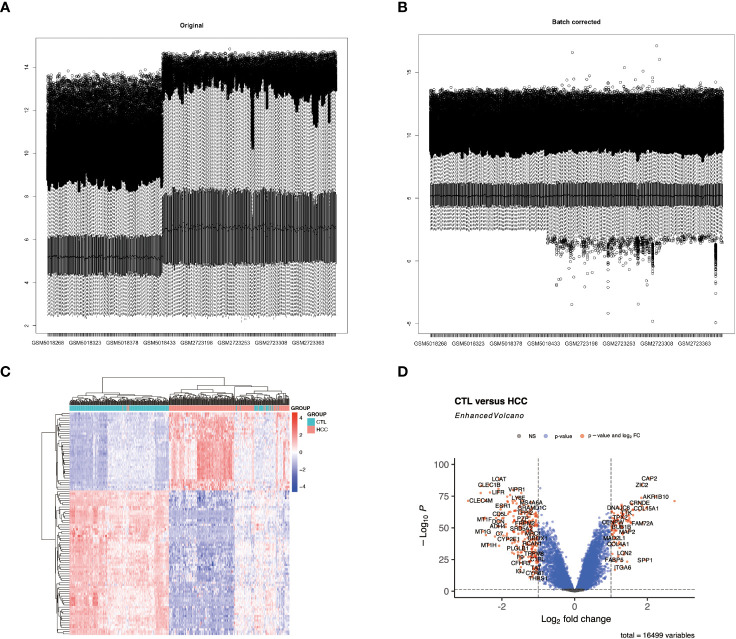
Differential expression analysis of the integrated HCC dataset. **(A, B)** display histograms of the batch effect corrected dataset. The x-axis represents the samples, and the y-axis represents the gene expression levels. **(C)** shows a heatmap of all differentially expressed genes (DEGs) in HCC. **(D)** features a volcano plot illustrating the DEGs in HCC.

The datasets related to DKD and HCC in the GEO database (https://www.ncbi.nlm.nih.gov/geo) were screened by the following criteria: Count value > 50; human, microarray, and gene count > 10,000. Among them, the GSE96804 dataset collected from DKD glomeruli, and the GSE164760 and GSE102079 datasets from HCC livers were used for subsequent analyses in this work. After removing the irrelevant samples, the data was parsed with the GEOquery R package (v2.66.0), and the ComBat method in the sva R package (v3.46.0) was used to adjust for bias across batches (see **Figures 1A, B**). R version 4.2.1 was used for this work.

### 2.2 Differentially expressed genes analysis

The DEGs in the DKD and HCC datasets were identified using the “ Limma “ package (version 3.54.0) in R. The screening thresholds for the DEGs in the DKD (**Supplementary Table S1**) and HCC (**Supplementary Table S2**) datasets were |log2 (fold change) | ≥ 1 and adjust p ≤ 0.05. The expression patterns of DEGs from HCC were then visualized in the form of volcano plots and heatmaps using the ggplot2 (version 3.4.0), EnhancedVolcano (version 1.16.0), and Pheatmap (version 1.0.12) R packages, respective.

### 2.3 Weighted correlation network analysis and key module gene isolation

WGCNA (version 1.70-3) was used to find cluster genes and their relation to external traits. Briefly, the median absolute deviation (MAD) was first calculated for each gene in the HCC dataset, and only the top 75% of genes were retained since low-expressed or non-varying genes usually represent noise. Next, scale-free co-expressed gene networks were constructed using the one-step network construction function of the “WGCNA” software package, with a soft threshold power (β = 5) as the weighting value. Then, after obtaining the module eigengenes (ME) for each cluster, the degree of association between MEs and features was calculated based on the association between MEs and clinical features. Finally, after screening, the most relevant MEs for HCC, GS, and MM measurements were used to identify genes highly associated with HCC (kME_MM>0.8), as well as associated members of the module (**Supplementary Table S3**). Genes differentially expressed in HCC in ME were determined as candidate genes associated with HCC (**Supplementary Table S4**).

### 2.4 Acquisition of possible secreted RNAs from DKD

We downloaded 17,534 extracellular vesicles (EVs) long RNAs from Exorbase (http://www.exorbase.org/, **Supplementary Table S5**). Considering the possibility of future non-invasive detection of blood markers, secreted proteins appearing in blood and urine were considered possible blood-based markers in clinical trials. Genes up-regulated and predicted to be secreted in the DKD dataset were then considered DKD secretory genes (**Supplementary Table S6**).

### 2.5 The construction of protein-protein interaction network

Since exosomal mRNAs are functional, recipient cells can take them up and translate them (17). To identify potential connections between DKD secretory RNAs and candidate genes associated with HCC, we constructed a protein-protein interaction (PPI) network using the STRING database (https://string-db.org/). Interrelationships between proteins were optimized with a medium confidence score > 0.4 (**Supplementary Table S7**). Then, the PPI network was loaded in Cytoscape (version 3.9.1) software and was split into community sub-networks using the leading eigenvector algorithm with the clusterMaker2. The top 3 large clusters of genes are considered to be DKD-driven HCC causative genes. The functional enrichment analysis was performed using clusterProfiler (version 4.8.2). During Gene Ontology (GO) and Kyoto Encyclopedia of Genes and Genomes (KEGG) enrichment analysis, pvalueCutoff = 0.05 or qvalueCutoff = 0.05 were chosen as filter parameters after pAdjustMethod was set to “BH,” respectively. The enrichment results for GO are shown in bubble charts, and the analysis results for KEGG are shown in circos plots.

### 2.6 Connectivity map analysis

In this study, we loaded the up-regulated genes from the PPI top 3 modules genes (**Supplementary Table S8**, 67 genes)into the CMAP database (https://clue.io) to screen for possible small molecule therapeutic agents. The top 10 compounds with the highest normalized connectivity scores (NCS) were identified as possible therapeutic agents (**Supplementary Table S9**). These molecules might down-regulate our input genes.

### 2.7 Machine learning screens for possible diagnostic genes

In order to identify candidate biomarkers and establish a DKD-related diagnostic model for HCC, the genes shared by DKD secretory genes, HCC DEGs, and the top 3 cluster genes are considered the possible Hub genes. **Supplementary Tables S2, S6**, and **S8** were used to screen the DKD-related HCC possible Hub genes. We used the “binomial” algorithm from the “glmnet” package (Version 4.1-4) to fit a LASSO regression model to screen candidate biomarkers. Next, the “randomForest” package’s non-linear nature (Version 4.6-14) was utilized to find candidate biomarkers expressed in HCC. We used the Boruta feature in machine learning to identify key categorical variables and determine fundamental mtry values. The best-trained model was used to screen the hub genes (**Supplementary Table S10**). The intersection of Lasso and randomForest results was considered the critical, pivotal genes for developing DKD-related HCC (**Supplementary Table S11**).

### 2.8 Construction of nomograms and ROC curves

ROC curves were plotted using the proc software package to assess the value of the four pivotal genes in HCC diagnosis. Then, nomograms for the four hub genes were constructed using the “rms” software (Version 6.7-1) package. Calibration curves and decision curve analysis (DCA) were further performed to assess the diagnostic value of the nomograms.

### 2.9 Immune infiltration analysis

The type and amount of immune cell infiltration were assessed from HCC expression profiles using the “CIBERSORT” software package (18). The Wilcoxon test compared the proportions of 22 immune cell types in HCC to control. The correlation of the four hub genes with these immune cell changes was further assessed. Finally, the degree of correlation between the 22 invading immune cells in HCC was shown using the “corrplot” software package (Version 0.84).

### 2.10 Patients’ samples collection

Blood samples from patients with DKD and DKD-HCC were obtained from the Fifth Hospital of Sun Yat-sen Hospital, China. Whole blood samples with EDTA were obtained from healthy controls (3), DKD patients (3), and DKD-HCC (3). The Ethics Committee of the Fifth Hospital of Zhongshan Hospital approved the protocol for collecting human samples.

### 2.11 qPCR

Human blood RNA was isolated using the MagJET(ThermoScientific). Amplification of target genes using a one-step RT-PCR system consisting of heat-stable SuperScript IV reverse transcriptase (Invitrogen) with high-fidelity Platinum SuperFi DNA polymerase(Invitrogen). The final volume of the reaction was 50 μL. For all experiments, refer to the instructions for generating amplifications of different lengths using gene-specific primers (**Supplementary Table S12**). The 2ˆ(-delta delta CT) method assessed gene expression. GAPDH was selected as the internal reference gene.

### 2.12 Statistical analysis

Experimental data are expressed as mean ± standard deviation. Differences between the two groups were generally compared using the unpaired Student’s t-test. p < 0.05 was considered statistically significant.

## 3 Results

### 3.1 Identification of DEGs in HCC

The combined HCC and control samples were analyzed for differences. A total of 346 DEGs were found, of which 103 were up-regulated and 243 down-regulated (**Figure 1**; **Supplementary Table S2**).

### 3.2 WGCNA analysis

**Figure 2:**
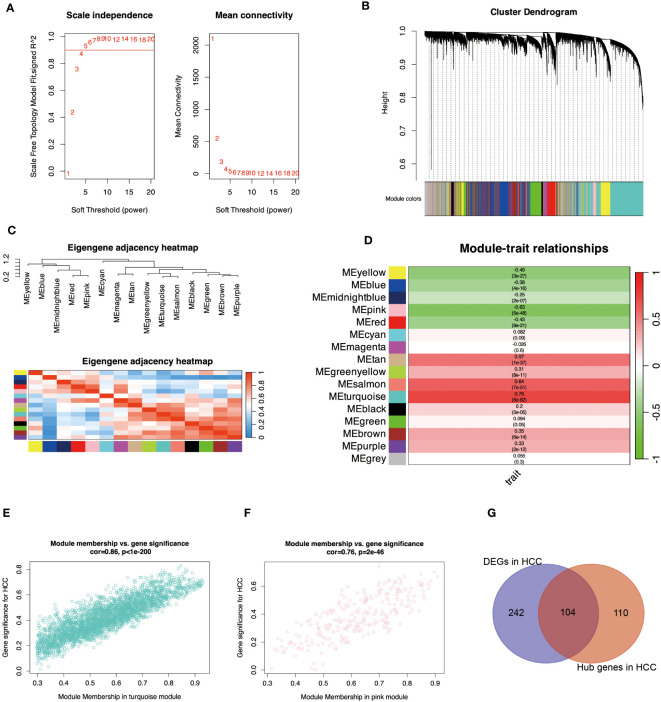
Identify essential module genes for HCC. **(A)** Determines the optimal β value using a scale-free topological model and selects β = 5 as the soft threshold based on average connectivity and scale independence. **(B)** Displays a hierarchical clustering dendrogram of the module identifiers. The dendrogram of genes was obtained by average chained hierarchical clustering—color rows below the dendrogram show module assignments determined by dynamic tree cuts. **(C)** Visually characterizes the correlation of the eigengenes. The branches (meta-modules) of the dendrogram combine sets of eigengenes that are positively correlated. The heatmap shows the neighbors in the Eigengenes network. Each row and column in the heatmap represent modular eigengenes (indicated by color). Blue indicates low adjacency with a negative correlation, and red indicates high adjacency with a positive correlation. **(D)** Displays a graph of the relationship between the module genes and the HCC. Each row corresponds to a module eigengenes and the column to a trait. Each cell was filled with the corresponding correlation and p-value. A redder color indicates a strong positive correlation between the phenotypic trait and the module eigengene, while a greener color indicates a strong negative correlation. **(E)** indicates the correlation between turquoise module members and the gene significance for HCC. Gene significance and module membership have a very significant correlation (0.86), implying that hub genes of the turquoise module also tend to be highly correlated with HCC. **(F)** indicates the correlation between the pink module members and the gene significance for HCC. Gene significance and module membership have a very significant correlation (0.76), implying that the hub genes of the pink module also tend to be highly correlated with HCC. **(G)** shows the intersection of crucial module genes with DEGs. The genes with R>0.5 from Module-trait relationships and kME_MM>0.8 in WGCNA analysis were considered hub genes in modules highly associated with HCC (**Supplementary Table S3**).

To discover key genes associated with HCC, we performed a WGCNA analysis using a scale-free topological fit index 5 to control for connected edges in the network (**Figure 2A**). **Figure 2B** shows the clustering of the genes, and **Figure 2C** shows the correlations between the modules. **Figure 2D** shows the correlations between these modules and the HCC, with turquoise color having the highest positive correlation with the HCC (r = 0.76) and pink having the highest negative correlation with the HCC (r = -0.63). Also, module membership and gene significance correlate highly with turquoise and pink modules in **Figures 2E, F**. The critical genes of DEGs and WGCNA in HCC samples were further intersected in **Figure 2G**, and 104 differentially expressed HCC-related genes were found (**Supplementary Table S4**).

### 3.3 Screening of DKD RNAs that may be secreted into the bloodstream

**Figure 3:**
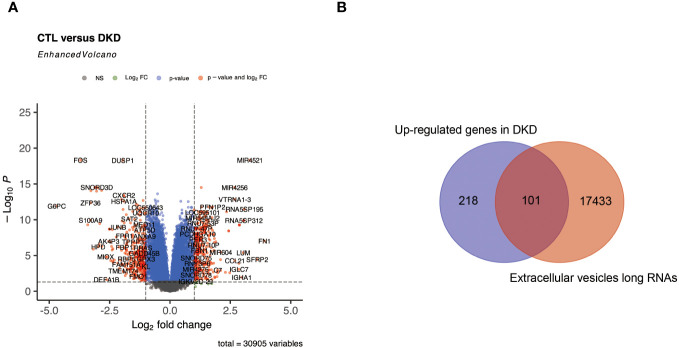
Identification of RNAs that may be secreted in DKD. **(A)** represents a volcano plot of DEGs in the DKD dataset. **(B)** represents a Venn plot of up-regulated genes and RNAs that may be secreted into the bloodstream in DKD.

We hypothesized that genes that have secretory properties and are upregulated in DKD may have the ability to influence HCC. In this work, we first isolated 661 DEGs from the DKD dataset (**Figure 3**), of which 319 genes were upregulated. Considering that these genes may contribute to the occurrence or development of HCC by releasing secreted RNAs, it was predicted from public databases that 101 of these RNAs may be secreted (**Supplementary Table S6**).

### 3.4 PPI analysis of DKD-associated HCC causative genes

**Figure 4:**
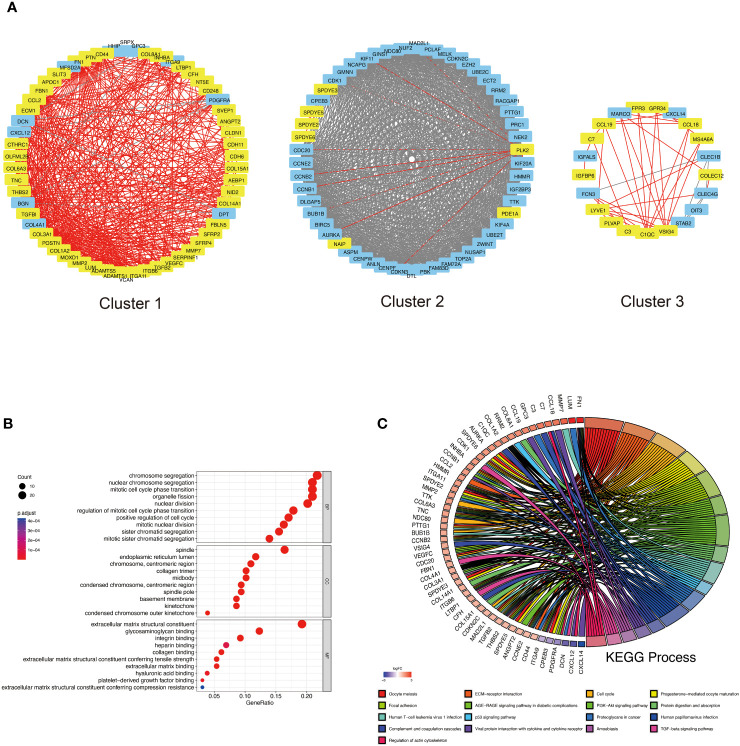
PPI analysis of DKD-related secreted genes and HCC-related genes. **(A)** Top 3 cluster genes were obtained based on the leading eigenvector algorithm. Yellow markers indicate possible DKD secretory genes and red lines are their interrelationships with other proteins. Blue genes are additional genes related to HCC in the PPI network, and the grey arrows represent their interrelationships. Red letters indicate genes belonging to DKD and HCC. **(B)** GO analysis of Top 3 cluster genes, including biological process (BP), cellular component (CC), and molecular function (MF). **(C)** indicates the circle plot of KEGG analysis results. Gene symbols with logarithmic values of logFC (blue-red scale) are located on the left side of the circos. The colored links on the right side link genes to KEGG annotations.

We constructed a PPI network using the STRING database to recognize the possible causative genes of DKD-related HCC from 101 up-secreted and 104 HCC-related DEGs. The Cytoscape software identified three important protein interaction modules with a leading eigenvector algorithm. These three modules contained 132 genes, 67 from DKD and 68 from HCC (**Supplementary Table S8**). Eight genes, COL15A1, ECM1, CTHRC1, C7, LUM, MS4A6A, PLVAP, and LYVE1 belong to DKD and HCC (**Figure 4A**). Their relationships with DKD and HCC gene interactions are displayed in **Supplementary Figure S1**. To understand the function of the causative genes, we analyzed them using GO and KEGG. In **Figure 4B**, chromosome segregation and nuclear chromosome segregation from Biological Process, spindle, and endoplasmic reticulum lumen from Cellular Component, extracellular matrix structural constituent, and glycosaminoglycan binding from Molecular Function were enriched. The Oocyte meiosis and ECM-receptor interaction were enriched in the KEGG enrichment analysis (**Figure 4C**). Interestingly, some critical pathways associated with inflammation were also enriched, such as the AGE-RAGE signaling pathway in diabetic complications, PI3K-Akt signaling pathway, TGF-beta signaling pathway, and viral protein interaction with cytokine and cytokine receptor.

### 3.5 Identification of candidate small molecule compounds that may reverse HCC

**Figure 5:**
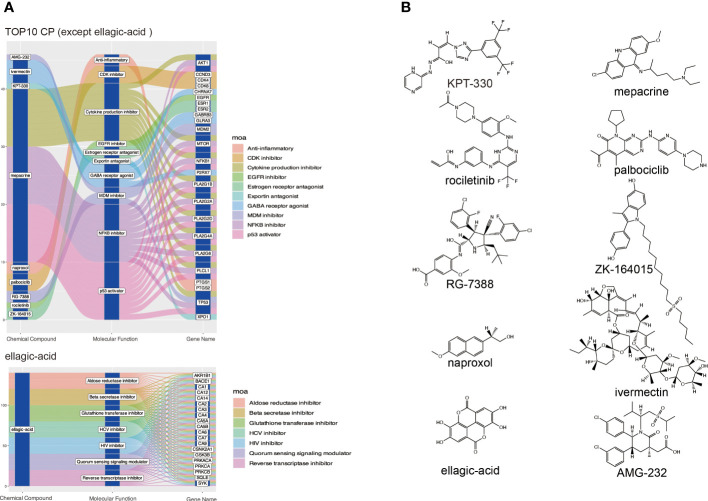
Screening potential small molecule compounds for DKD-related HCC by cMAP analysis. **(A)** The top 10 compounds with the highest negative enrichment scores based on cMAP analysis, along with their targets and the cellular pathways that may be affected. **(B)** Chemical structures of the ten compounds.

To explore small molecule drugs that may reduce genes in patients with DKD-related HCC. We used the up-regulated genes in the top 3 clusters (**Supplementary Table S3**) to screen drug-treated cells with similar expression patterns to predict potential small-molecule drugs that may have a therapeutic effect on DKD-associated HCC patients. After cMAP screening, there were ten compounds with the highest negative scores, including KPT-330, rociletinib, RG-7388, naproxol, ellagic-acid, mepacrine, palbociclib, ZK-164015, ivermectin, and AMG-232 were considered potential pharmacotherapeutic agents for the treatment of DKD-related HCC. **Figure 5** illustrates these ten compounds’ targeted cellular pathways, target genes, and chemical structures. The targets of some of these molecules, including NFKB, P53, EGFR, and cytokine, are highly associated with inflammation.

### 3.6 Screening hub genes with diagnostic value by machine learning

**Figure 6:**
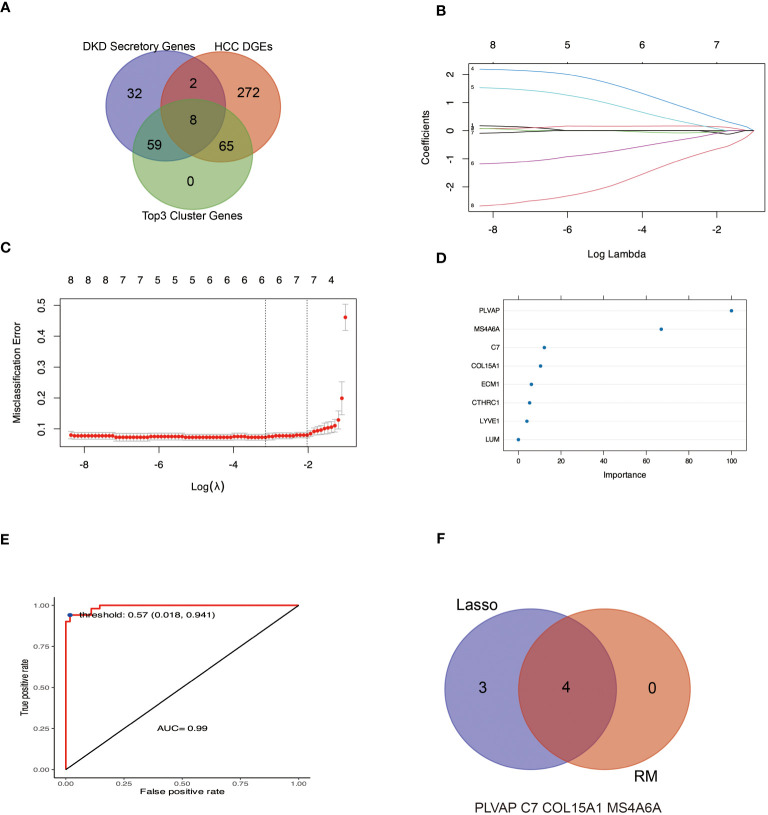
Machine learning methods were used to identify hub genes for DKD-related HCC. **(A, B)** The LASSO logistic regression algorithm was used to identify seven possible markers of HCC. **(C, D)** indicate the genes characterized by RF to determine the importance genes of HCC, of which four genes had MeanDecreaseGini greater than 10. **(E)** Based on the selected optimal threshold, the model’s prediction accuracy was evaluated by a confusion matrix. **(F)** The Venn diagram shows four genes in common with the LASSO and RF algorithms.

Considering that DKD secretory RNAs and HCC DEGs can form 3 key PPI groups, coupled with the fact that these genes are up-regulated in both DKD and HCC and have a high rate of correct diagnosis of HCC, it can be assumed that these genes are the pivotal genes for DKD-associated HCC. In this work, we utilized a Lasso and random forest (RF) machine learning algorithm to isolate the four hub genes from the candidate gene (**Figure 6**). Plasmalemmal Vesicle-Associated Protein (PLVAP), complement C7(C7), collagen type XV alpha 1 chain (COL15A1) and membrane spanning 4-domains A6A (MS4A6A) were recognized as the hub genes.

### 3.7 Construction and efficacy assessment of diagnostic nomogram models

**Figure 7:**
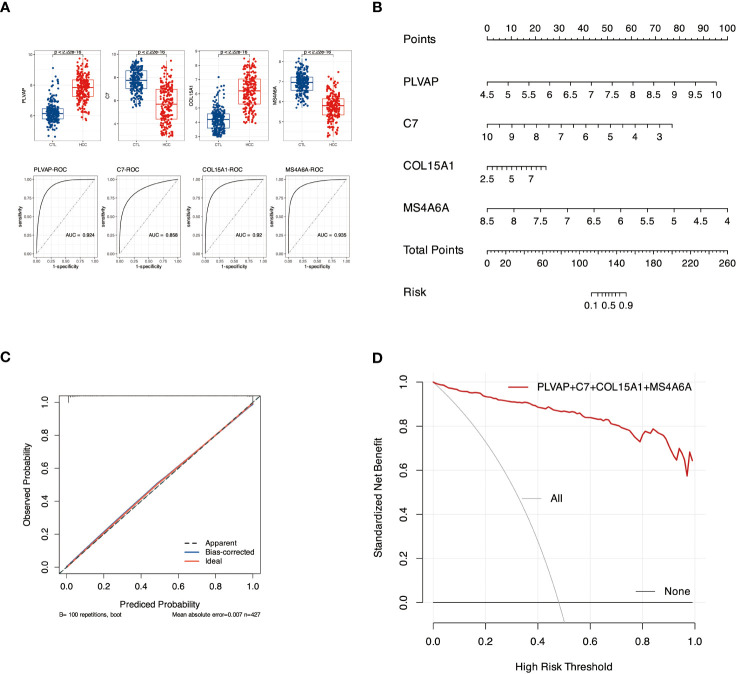
Diagnostic nomogram model construction and efficacy assessment. **(A)** Expression and ROC profiles of candidate biomarkers, PLVAP, C7, COL15A1, and MS4A6A, between control and pathogenic groups. **(B)** The nomogram model. **(C)** The calibration curve of nomogram model. **(D)** denotes DCA for the nomogram model. The black line labeled “none” represents the net benefit of assuming no patients had HCC. The gray line labeled “all” means the net gain assuming all patients have HCC, and the red line labeled genes represents the net profit bearing DKD-related HCC is identified based on the HCC diagnosis values predicted by the nomogram model.

We first evaluated the efficacy of PLVAP, C7, COL15A1, and MS4A6A as sample classifiers for better diagnosis and prediction. The Area Under Curve (AUC) of the four genes in **Figure 7** A was more significant than 0.85. Based on this, we constructed a diagnostic nomogram model with these four genes (**Figure 7B**) and validated the predictive efficacy of the model using Bootstrap self-sampling (**Figure 7C**). In addition, the Decision Curve Analysis (DCA) curves (**Figure 7D**) showed that our fit curve was far from the two extreme curves, implying that the model has application value.

### 3.8 Correlation analysis of immune cell infiltration and core genes in HCC

**Figure 8:**
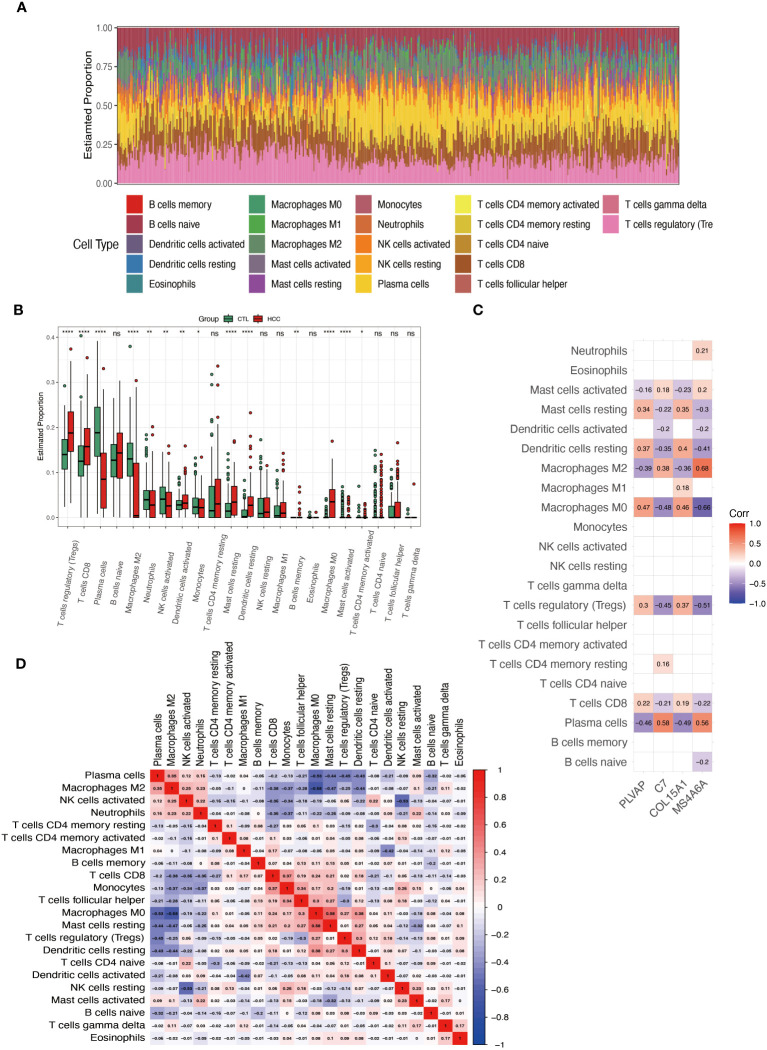
Analysis of immune cell infiltration in HCC. **(A)** Superimposed histogram shows the proportion of immune cells in the HCC and control groups. **(B)** Bar graph compares the 22 immune cells in the HCC and control groups. **(C)** Heatmap shows the correlation of the genes PLVAP, C7, COL15A1, and MS4A6A with the infiltration of the 22 immune cells. **(D)** Heatmap showing the correlation of 22 immune cell infiltrations. *p < 0.05; **p < 0.01; ****p < 0.0001; ns, not significant.

Our PPI results showed that the genes in the top 3 clusters were associated with the AGE-RAGE signaling pathway in diabetic complications, the TGF-beta signaling pathway, and cytokines. These pathways are all related to inflammation. Therefore, analyzing the immune cells of HCC with the CIBERSORT algorithm is applicable further to understanding the four hub genes involved in DKD-related HCC. **Figure 8A** shows the proportion of 22 immune cells in each sample. **Figure 8B** demonstrates the differentiation of these 22 cell types in HCC. Compared with the control group, T cells regulatory (Tregs), T cells CD8, Plasma cells, Macrophages M2, Mast cells resting, Dendritic cells resting, Macrophages M0, Mast cells activated have significant changes. MS4A6A (**Figure 8C**) shows the highest correlation with Macrophages M2 (r=0.68) and the highest negative correlation with Macrophages M0 (r=-0.66). **Figure 8D** shows that Macrophage M0 and M2 are highly negatively correlated (r=-0.68), while Plasma cells are negatively correlated with Macrophage M0 (r=0.-53).

### 3.9 Validation of core gene expression in the blood of DKD patients

**Figure 9:**
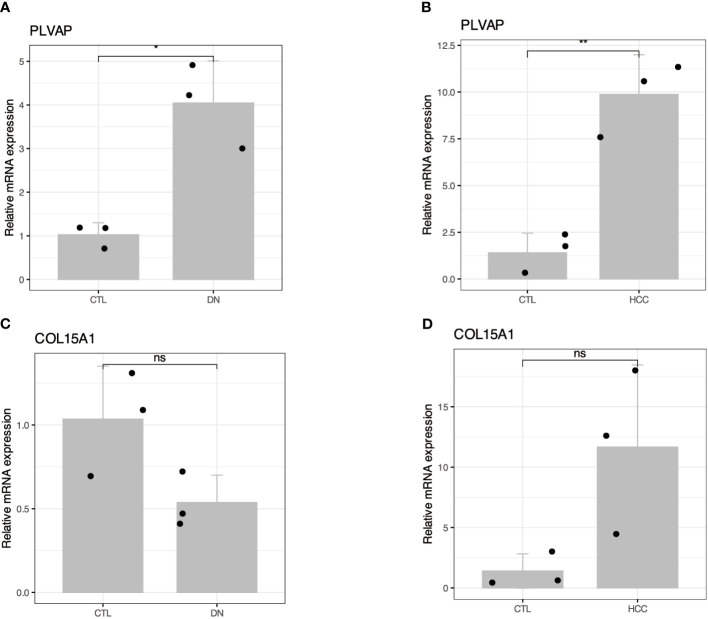
Validation of the expression patterns of two hub genes in DKD and DKD-HCC comorbidity. **(A, B)** RT-qPCR showed increased mRNA levels of PLVAP in DKD and DKD-HCC comorbidity, respectively. **(C, D)** RT-qPCR showing altered mRNA levels of COL15A1 in DKD and DKD-HCC comorbidity. The value of 2^–ΔΔCT^ is the relative expression of mRNA from each gene compared with GAPDH. The CT value is the number of fractional cycles at which a fluorescent signal passes a fixed threshold. *p < 0.05; **p < 0.01; ns, no significance.

The high expression of PLVAP and COL15A1 genes in the blood of DKD patients may be a critical activator for DKD leading to HCC. To further confirm the accuracy of the comprehensive bioinformatics analysis described above, we examined the expression of PLVAP and COL15A1 in the blood of patients with DKD and DKD-HCC using qPCR. **Figure 9** shows that PLVAP was highly expressed in DKD and DKD-HCC patients.

## 4 Discussion

We are the first to screen key genes for DKD that may cause HCC by applying multiple bioinformatics and machine learning analysis methods. Our results showed that the AGE-RAGE signaling pathway in diabetic complications, PI3K-Akt signaling pathway, TGF-beta signaling pathway, P53 signaling pathway, and other signaling pathways might associated with DKD-related HCC. Using machine learning, we isolated four genes, PLVAP, C7, COL15A1, and MS4A6A, as the hub genes that DKD may affect subsequent HCC. By confirming the ROC curves, we used these four genes to build a diagnostic nomogram model for DKD-related HCC. Finally, we confirmed that PLVAP can be used as a blood RNA marker to diagnose DKD or DKD-HCC, and the role of this gene may be used to assess the risk of HCC in DKD patients.

Chronic kidney disease (CKD) is a significant public health problem worldwide, and patients with CKD appear to be at higher risk of developing HCC compared to the general population (10). DKD is the most common form of Chronic Kidney Disease (CKD) and has the highest prevalence of End Stage Kidney Disease (ESKD) worldwide (19, 20). Recent studies have shown that the pathophysiology of DKD is multifaceted and that DKD has been characterized as a metabolically driven immune disorder. Numerous studies have shown that inflammation leads to deterioration of kidney function. High-Sensitivity C-Reactive Protein is a systemic marker of inflammation associated with the progression of DKD in patients with T2DM (21). Not only that, the Systemic immune-inflammation index (SII), an index calculated by platelet count × neutrophil count/lymphocyte count, was used to assess T2D-associated DKD (19). Interestingly, the SII index has been initially used to assess the prognosis of patients with HCC by Hu et al. (22). It is unclear why the immune indices in blood are suitable for the diagnosis or prognosis of DKD and HCC. The potential inflammation factors and mechanisms participating in DKD-related HCC are not fully understood.

HCC is the second leading cause of cancer deaths globally and has multiple etiologic factors, most of which are related to inflammation (23).

Telomere shortening and consequent chromosomal instability are observed in 90% of HCC carcinogenesis and progression due to increased hepatocyte proliferation (24). At the subcellular level, there is a high frequent impairment of the spindle assembly checkpoint in HCC (25), and the accumulation of misfolded and unfolded proteins in the lumen of the endoplasmic reticulum (ER), which induces ER stress and leads to activation of the unfolded protein response (26). Recent research has reported that a 2–3 fold increase in heparan sulfate N-sulfation/O-sulfation ratio was observed in HCC compared to cirrhotic tissues (27), indicating the expression of glycosaminoglycans in cirrhotic liver and HCC are different. These reported impaired Biological Process, Cellular Component, and Molecular Function pathways are entirely consistent with our work’s GO enrichment analysis of DKD-driven HCC causative genes (**Figures 4A, B**). It is worth noting that there is justification for using DKD-secreted RNA as a seed for PPI network analysis (**Figure 4A**). Exosomes can fuse with the plasma membrane of target cells, delivering mRNA material to the cytoplasm for post-translational interactions with proteins (28). In this study, the hypothesis was that a portion of the exosomal RNA could be intercepted by cells in the bloodstream (such as inflammatory cells) and then migrate to the liver, becoming part of the HCC immune infiltrate. Another portion of the RNA may directly target liver tissues, where it can be translated and interact with other proteins within hepatocytes. However, these mechanisms were not confirmed in this study. Additionally, HCC is a classic example of inflammation-associated cancer, as more than 90% of HCCs arise in the context of liver injury and inflammation (29). Several signaling pathways, including TGF-β, ECM, AGE-RAGE, PI3K-AKT, P53 and cytokines, are dis-regulated in HCC and lead to uncontrolled cell division and metastasis (30–33). Consistent with the reported impaired KEGG pathway, our KEGG enrichment analysis showed that most of the causative genes of DKD-associated HCC were enriched in inflammatory and immune-related pathways, suggesting that the inflammatory-immune pathway may be a potential mechanism of DKD-associated HCC (**Figure 4C**).

Even though HCC is one of the deadliest health burdens worldwide, few drugs are available for clinical treatment (34). First-line therapeutic agents include sorafenib and levatinib. The former is a multikinase inhibitor that blocks the activity of RAF-1, BRAF, VEGFR, PDGFR, and KIT receptors involved in cell proliferation and angiogenesis (35). The latter targets VEGFR 1-3, FGFR 1-4, PDGFR α, RET and KIT, and is a first-line systemic treatment for advanced HCC (36). Both of them can contribute to antitumor activity with Immunomodulatory activity (37, 38). There have been no more in-depth explorations to distinguish the differences in clinical benefit of these agents for HCC and DKD-related HCC. Our study provides a new perspective by linking pathogenic genes associated with DKD through cMAP analysis to identify potential compounds targeting HCC. This work applied possible DKD-related pathogenic genes up-regulated by HCC to the cMAP analysis. Ten small molecule compounds were identified (**Figure 5**). Among them, mepacrine, originally used as an antimalarial for nearly a century, has recently been rediscovered as an anticancer drug (39), based on its function in inhibiting the NFκB pathway and inducing p53 expression. Interestingly, like Mepacrine, the ellagic-acid, which has antiviral effects, has also been shown to have applications in the treatment of HCC (40), shows high against inflammation activities with a prolonged onset and duration through interaction with known cyclooxygenase inhibitors (41). Based on these findings, Mepacrine and ellagic-acid may be consistent with first-line drugs for treating HCC but are more applicable to DKD-associated HCC for their lower norm_cs score (**Supplementary Table S10**). It can be hypothesized that the early use of anti-inflammatory interventions in patients with DKD may improve renal function, inhibit the onset and progression of HCC, and ultimately significantly prolong the life span of patients.

AFP is a biomarker widely used for HCC detection and disease surveillance, and most experts believe that AFP does not have sufficient performance characteristics to serve as a standalone screening test (42). Even the AFP-L3, a fucosylated glycoform of AFP, is insufficient as a standalone test for screening. In an independent phase III cohort of 534 patients in the United States, the cutoff value for AFP-L3 was 8.3%, the sensitivity for early HCC was 40%, and the FPR was fixed at 10% (43). There is a lack of effective biomarkers that can be used for early HCC detection. In our work, we identified four signature genes using machine learning from eight causative genes that may be associated with DKD-related HCC. These four genes, PLVAP, C7, COL15A1, and MS4A6A were then used to construct a diagnostic nomogram model that could effectively diagnose DKD-associated HCC (**Figure 7**). Furthermore, we chose the genes PLVAP and COL15A1, which are highly expressed in both DKD and HCC, to facilitate the detection as blood-based biomarkers for HCC. Although the human samples from GSE96804 were ethnically different from our blood samples, PLVAP expression was up-regulated in blood with both DKD and DKD-HCC comorbidities, consistent with the results of the bioinformatics analysis. The PLVAP protein is the main component of endothelial diaphragms in fenestrae, caveolae, and transendothelial channels, shown in previous studies to be associated with DKD and HCC. On the one hand, PLVAP can be used as an early marker of glomerular endothelial injury with DKD in mice (44), and do increase in glomeruli of human diabetic patients (45). On the other hand, PLVAP was identified as a gene expressed explicitly in HCC vascular endothelial cells (46), and has been investigated as a therapeutic target in HCC (46), perhaps based on its ability to alter the immunosuppressive microenvironment (47). Indeed, during inflammation, PLVAP is required for leukocyte exudation into the site of inflammation and is essential for transcellular migration (48, 49), and has also been described as a leukocyte transport molecule that plays a crucial role in immune surveillance and inflammation as it is redistributed in cells after pro-inflammatory stimuli (50). The conclusion can be drawn that PLVAP may provide a potential immune-related diagnostic indicator for patients with DKD-related HCC.

Studies have highlighted that immune cell infiltration is essential in the study of HCC development and immunotherapy (51–54). Macrophages, thought to be an evolutionarily ancient cell type involved in tissue homeostasis and immune defense against pathogens, are now being rediscovered to act as modulators of various diseases, including cancer (55). Alternately activated (M2) macrophages promote tumor growth and invasiveness in HCC (56), and stimulate HCC cell migration and epithelial-mesenchymal transition through the TLR4/STAT3 signaling pathway (57). In this work, we found significant differences in immune cell infiltration between the HCC and control groups, including T cells regulatory (Tregs), T cells CD8, Plasma cells, Macrophages M2, Mast cells resting, Dendritic cells resting, Macrophages M0, Mast cells activated. The Macrophages M2, M0, and Plasma cells were associated with PLVAP, C7, COL15A1, and MS4A6A. Since MS4A6A has been reported as a biomarker for macrophage M2 (58), it gives the highest correlation with macrophage M2. The other three hub genes (**Figure 8C**) also showed significance in correlation with macrophage M2 and M0, suggesting that these four genes used to construct nomograms may influence the development of DKD-associated HCC through interactions with immune cell infiltration.

## 5 Conclusion

Through the combined analysis of RNAs that may be secreted by DKD and gene clusters associated with HCC, we revealed the inflammatory immune pathways of DKD that may affect HCC. Based on these pathways, we screened small molecule drugs that can be used to treat DKD-related HCC. Then, utilizing bioinformatic and machine-based approaches such as ROC, lasso, and RM, we screened PLVAP, C7, COL15A1, and MS4A6A to construct HCC diagnostic nomograms. After thoroughly evaluating the diagnostic efficacy of the four genes, we assessed the potential of PLVAP as a blood-based biomarker for disease diagnosis in blood samples of DKD and DKD-HCC. Our work provides new insights into using immune pathway molecules to diagnose and treat DKD-related HCC.

## Data availability statement

The datasets presented in this study can be found in online repositories. The names of the repository/repositories and accession number(s) can be found in the article/**Supplementary Material**.

## Ethics statement

The studies involving humans were approved by The Fifth Affiliated Hospital of Sun Yat-sen University. The studies were conducted in accordance with the local legislation and institutional requirements. The participants provided their written informed consent to participate in this study.

## Author contributions

CC: Data curation, Funding acquisition, Investigation, Methodology, Validation, Writing – original draft. ZX: Resources, Writing – review & editing. YN: Data curation, Investigation, Methodology, Writing – review & editing. YH: Data curation, Investigation, Methodology, Supervision, Writing – review & editing.

## Supplementary material

Supplementary Figure 1 PPI network of 8 common genes of DKD and HCC. Light blue rectangles indicate candidate RNAs secreted by DKD, yellow indicates the eight genes shared by DKD and HCC, and blue indicates the genes associated with HCC. Red lines show the relationship between the eight genes and other genes; gray lines indicate the relationship between other genes.
