# Supplementary figures and images for: Screening immune-related blood biomarkers for DKD-related HCC using machine learning
Source: Front Immunol. 2024 Jan 22;15:1339373. doi: 10.3389/fimmu.2024.1339373 (PMC10838782; doi:10.3389/fimmu.2024.1339373)

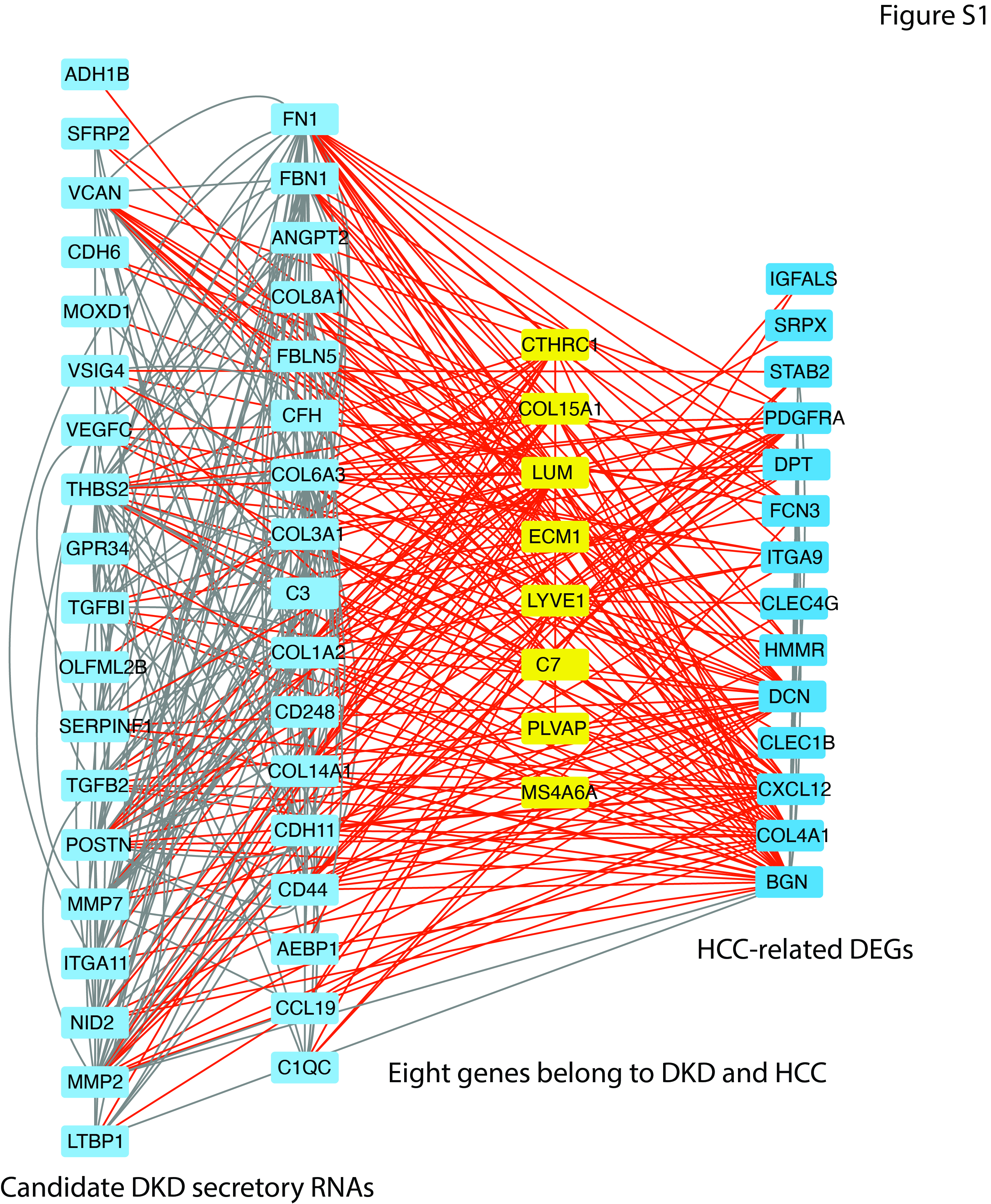

Supplement: Supplementary Figure 1 — PPI network of 8 common genes of DKD and HCC. Light blue rectangles indicate candidate RNAs secreted by DKD, yellow indicates the eight genes shared by DKD and HCC, and blue indicates the genes associated with HCC. Red lines show the relationship between the eight genes and other genes; gray lines indicate the relationship between other genes. [file Image_1.jpg]
